# Microbial Infections and Wound Healing: Medicinal-Chemistry and Technological Based Approaches

**DOI:** 10.3390/pharmaceutics16020168

**Published:** 2024-01-25

**Authors:** Ivana Cacciatore, Lisa Marinelli

**Affiliations:** Department of Pharmacy, “G. d’Annunzio” University of Chieti-Pescara, 66100 Chieti, Italy; l.marinelli@unich.it

Microbial infections represent a significant global health challenge that impacts all populations [1]. Addressing this worldwide problem requires coordinated global efforts. Notably, wound infections represent one of the major clinical causes of morbidity and mortality [2]. Damaged tissues are more vulnerable to microbial infections, which induce alterations and may delay the wound-healing process [3,4]. In this regard, ineffective wound management has great social and economic impacts on the patients’ quality of life. Fortunately, in recent years, scientific communities have introduced new and attractive perspectives of wound healing [5]. Addressing microbial infections in wounds requires a comprehensive and multidisciplinary approach [6,7,8].

This Special Issue delves into medicinal chemistry related to the development of new antimicrobial agents, as discussed by Turkez H. et al. and Amariucai-Mantu D. et al. Specifically, Turkez et al. investigated the use of alpha-lipoic acid (ALA)-conjugated hexagonal boron nitride (hBN) and boron carbide (B4C) nanoparticles (NPs) to improve wound healing in human dermal fibroblast cells and assess their antimicrobial properties against *S. aureus* and *E. coli*. Their findings showcased that ALA-conjugated hBN NPs exhibited superior wound-healing features and antimicrobial activity compared to those of ALA-B4C. The conjugation of ALA notably enhanced the wound healing and antimicrobial effectiveness of both the hBN and B4C NPs. Amariucai-Mantu D. et al. reported the advances in the field of hybrid azine derivatives with antimicrobial activity over the last five years. They showed that the combination of an azine moiety with a five-member ring azaheterocycle resulted in the best approach to obtain drugs with improved and superior antimicrobial properties. Based on their studies, hybrid azine merged with a five-member ring azaheterocycle appears to be one of the most promising fields of research within this area.

Currently, the efforts are also focused on the development of innovative therapeutic strategies regarding drug delivery systems for treating microbial infections and acute/chronic wounds [9,10]. Drug delivery systems in antimicrobial therapy represent an innovative approach to optimize the efficacy and safety of antimicrobial agents [11]. These systems aim to optimize the pharmacokinetics and pharmacodynamics of antimicrobial drugs, ensuring controlled release and sustained therapeutic levels at the infection site. The overarching goal is to enhance the therapeutic outcomes, while minimizing the side effects and toxicity [12]. In this Special Issue, Coleman L. et al. focused on the antibacterial and antibiofilm capabilities of several non-antibiotic compounds (polyhexamethylene biguanide (PHMB), curcumin, retinol, polysorbate 40, ethanol, and D-α-tocopheryl polyethylene glycol succinate 1000 (TPGS)) formulations. PHMB was observed to have highly effective antibacterial activity against both *S. aureus* and *P. aeruginosa*. Meanwhile, TPGS had a limited inhibitory activity, but demonstrated potent antibiofilm properties. The combination of these two compounds in a formulation resulted in the synergistic enhancement of their capability to kill both *S. aureus* and *P. aeruginosa* and disperse their biofilms.

Nisin-loaded PLGA nanoparticles were developed by Üstün A. et al. to treat the infections caused by *S. aureus*, while Marinelli L. et al. reported the wound-healing and anti-inflammatory properties of carvacrol prodrugs/hyaluronic acid formulations. In the first paper, the data suggest the potential application of the corresponding formulation as a bioactive tool to regulate the wound repair process by inhibiting microbial infections. In the second study, the data suggest that the developed formulations may be applied to modulate the inflammatory and remodeling phases, affecting the activity of the immune cells. Also, Falbo F. et al. delved into the topic of natural compounds, focusing on biopolymer-based hydrogels to promote the wound-healing process. Novel methods and devices for efficient wound care in individuals with diabetes were well documented and thoroughly examined.

Biocompatible ciprofloxacin–gold nanoparticle-coated sutures for surgical site infections were developed by Sampathi S. et al., showing that the use of antibiotic-coated sutures for preventing surgical site infections for a long duration could be a viable clinical option to the current alternatives.

In conclusion, this Special Issue provides a comprehensive overview of microbial infections, with a specific focus on wound-healing strategies. These studies highlight the evolving field of medicinal chemistry, showcasing the development of novel antimicrobial agents and innovative drug delivery systems. The diversity of these approaches, from exploring natural compounds to engineering biocompatible materials, demonstrates the interdisciplinary efforts aimed at optimizing the therapeutic outcomes.

As we navigate the global health challenge posed by microbial infections, the findings presented in this Editorial underscore the importance of a multifaceted and collaborative approach. The insights from these studies contribute not only to our understanding of wound-healing process, but also to the potential development of effective treatments for microbial infections. The progress in this field is promising, offering hope for improved patient outcomes and a reduced social and economic burden associated with ineffective wound management.

Moving forward, continued research and collaboration among scientific communities will be crucial to advancing our knowledge and translating these innovations into practical solutions for better wound care. The integration of diverse strategies, as highlighted in this Editorial, introduces new avenues for the future of antimicrobial therapy and wound healing.
